# Bone and joint infections with *Staphylococcus aureus* strains producing Panton–Valentine Leukocidin in French Guiana

**DOI:** 10.1097/MD.0000000000016015

**Published:** 2019-07-05

**Authors:** Coralie Hardy, Lindsay Osei, Thierry Basset, Narcisse Elenga

**Affiliations:** Department of Pediatric Medicine and Surgery, Cayenne Hospital, Cayenne Cedex, French Guiana.

**Keywords:** bone and joint infections, clindamycin, French Guiana, Panton–Valentine Leukocidin producing *Staphylococcus aureus*, pericarditis

## Abstract

The aim of this study was to describe the clinical features of bone and joint infections (BJI) due to Panton-Valentine Leukocidin producing (PVL+) *Staphylococcus aureus* (*SA*) in French Guiana.

A multicenter study that consists of a retrospective charts review of children admitted for PVL+ *S. aureus* BJI between January 2010 and December 2015.

Six patients with *SA*-PVL BJI were identified during the study period: 2 osteomyelitis, 1 septic arthritis, and 3 disseminated BJI. The median age was 11 years old (4–14 years), and fever lasted for 3.2 days (2–5 days) before diagnosis. An open skin wound preceded the BJI in 5/6 patients. One patient presented with a septic thrombophlebitis of the femoral-popliteal vein on admission. Methicillin-susceptible *Staphylococcus aureus* (MSSA) were identified for all patients. Three patients had complications: 2 cases of necrotizing pneumonia and 2 pericarditis, with 1 death caused by cardiac tamponade.

*SA-*PVL BJI was not frequent. Strains were susceptible to methicillin, but responsible of severe BJI. Early diagnosis and a multidisciplinary management of these infections are essential to prevent further complications.

## 1 Introduction

Despite advances in the understanding and management of pediatric bone and joint infections (BJI), these infections continue to pose a diagnostic challenge to clinicians.^[1]^ Methicillin-susceptible *Staphylococcus aureus* (MSSA) is the first cause of BJI in children in French Guiana.^[2]^ Septic arthritis (SA) and osteomyelitis (OM) can rapidly become life-threatening infections and cause devastating functional impairment if they are not adequately and immediately treated.^[3]^ High morbidity and mortality is explained by *Staphylococcus aureus* (*SA*) extracellular virulence factors such as Panton–Valentine Leukocidin (PVL).^[4,5]^ PVL is a 2 leukocidin components, which causes a pore formation in the cytoplasmic membranes, resulting in a cytolytic efflux of molecules and metabolites.^[6]^ An increased incidence of bone and joint infections due to MSSA-PVL strains has been described over the last decade.^[7–9]^ However, the prevalence of PVL-associated BJI in children in South America has scarcely been reported.^[2]^

French Guiana is a French overseas department in northeastern Amazonia, located between Brazil to the east and Surinam to the west. In this tropical and isolated area, access to health care is often delayed due to a series of issues for part of the population: lack of means of transportation, difficult access to medical infrastructures, neglected skin infections. Tropical climate as well as poor housing and a community lifestyle should also be taken into account when studying the epidemiology of these community acquired infections. The objective of this study was to describe the clinical features of pediatric BJI due to *SA*-PVL strains.

## 2 Patients and methods

### 2.1 Study population

We conducted a retrospective charts review of all children <16 years of age, in all 3 hospitals of French Guiana. Their discharge code, according to the International Classification of Diseases (ICD), was consistent with the diagnosis of MSSA-producing PVL acute BJI, in the period from January 1, 2010 until December 31, 2015.

### 2.2 Definitions

Acute BJI was defined as any osteoarticular infection presenting with a time period between diagnosis and symptom onset <2 weeks.

OM was defined as the presence of clinical features (fever, pain, restriction of movement), radiological study that identified the location of the infection (bone scan, magnetic resonance imaging [MRI], computerized tomography [CT] or ultrasound), and with or without bacterial isolation from blood or bone sample.^[9]^

SA was defined as the presence of clinical features (fever, joint swelling, functional disability or pain), joint effusion demonstrated by ultrasound or by physical examination, and bacterial isolation from joint fluid or blood culture.^[9]^ Osteoarthritis (OA) was when the disease met criteria of both OM and SA, according to the above definitions.^[9]^

The following information were extracted from the patients’ charts:

- *Clinical data:* Clinical presentation, medical history, complications on admission and during hospital stay, long-term outcomes.
- *Imaging data*: X-ray, ultrasound, echocardiography, CT, MRI, and scintigraphy were collected for all patients when available.
- *Microbiological data:* These data came from the analysis of blood cultures, pus and sputum cultures. Identification of *SA* was obtained in French Guiana. Strains were sent for identification of PVL to reference centers for staphylococcal toxemia in Lyon or Strasbourg.
- *Treatment*: Information regarding antibiotics received by patients as well as surgery, when performed, were retrieved.
- *Ethical consideration:* Patients’ medical records were retrospectively reviewed, and all data collected were anonymized according to the French national committee—Commission Nationale de l’Informatique et des Libertés (*CNIL*)—that oversees research data. Moreover, all participants’ legal guardians had signed informed consent allowing medical and surgical treatment.

## 3 Results

### 3.1 Clinical features

Six cases of bone and joint infections due to MSSA-producing PVL were reported: 2 acute hematogenous osteomyelitis, 1 septic arthritis, and 2 disseminated osteoarticular infections. The median age was 11 years old (4–14 years). They had for 3.2 days (2–5 days) before consulting. An open skin wound preceded the BJI in 5 out of 6 patients. All patients presented with signs of local inflammation (swelling and pain) and limitation of movement or limping. Infections involved bones and joints of the lower extremities. Patients’ clinical features are summarized in Table 1. Our findings were compared with other case report studies (Table 2).

**Table 1:**
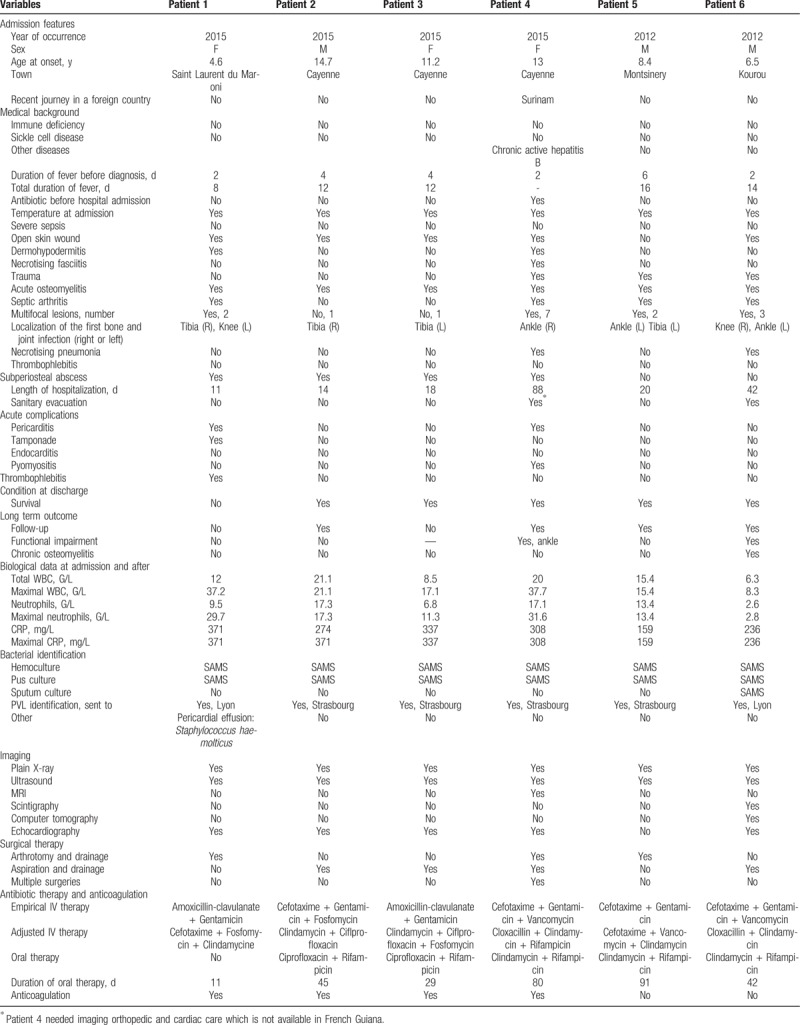
Summary table of clinical characteristics, laboratory, and long-term outcome of 6 PVL-SA BJI patients.

**Table 2:**
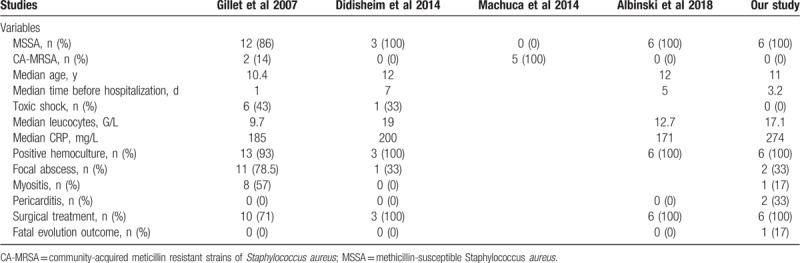
Comparison of patients’ characteristics from 4 previously published PVL-SA BJI studies and the current series.

### 3.2 Predisposition and risk factors

We did not identify any somatic predisposing conditions in our 6 patients. No patient had sickle cell disease and all patients had no immune deficiency and were healthy before the occurrence of this infection.

### 3.3 Laboratory findings

The median C-reactive protein (CRP) on admission was 274 mg/L (159–371). CRP was at its highest on admission for all patients. Five patients had leukocytosis with polymorphonucleosis.

### 3.4 Imaging results

All patients had a plain radiography after admission, and an injected MRI. Five out of 6 children had an echocardiography: 2 pericarditis were confirmed. One patient underwent a scintigraphy in Martinique, to identify multiple lesions. Four children with acute osteomyelitis had a subperiosteal abscess.

### 3.5 Surgical interventions

**Figure 1:**
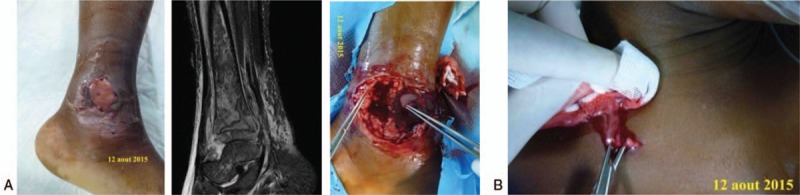
(A) Acute hematogenous osteomyelitis and ankle septic arthritis, before and during surgery. MRI, sagittal plane, STIR. (B) Pyomyositis.

All patients underwent surgery. A surgical drainage by arthrotomy was performed for 2 children: knee and ankle septic arthritis. Needle aspiration and drainage was needed for the other 4 patients who suffered from acute osteomyelitis (Fig. 1A and B).

### 3.6 Microbiological results

The isolation of a microorganism was possible in all cases. All 6 cases had a blood culture that came back positive for MSSA within 24 to 48 hours. In 3 out of 6 cases, the pus culture was positive for MSSA. One patient with a necrotizing pneumonia had a positive sputum culture for MSSA. The median time of negativization of blood cultures was 10 days.

### 3.7 Treatments

Empirical intravenous therapy including an antibiotic active against MSSA was started on admission. Two children received amoxicillin-clavulanate combined to gentamicin. Three children received cefotaxime combined to gentamicin. One child received ceftriaxone and amikacin. Clindamycin, that was shown to have an in vitro antitoxin effect, completed the therapy within 3 days for all patients. Subsequent oral therapy consisted of either ciprofloxacine and rifampicin, or clindamycin and rifampicin. Duration of oral therapy was of ≥6 weeks.

### 3.8 Follow-up and outcome, complications, and sequelae

Apyrexia was obtained for all patients within a mean of 12 days (8–16). One patient presented on admission with a septic thrombophlebitis of the femoral-popliteal vein. Three patients had secondary visceral complications: 2 cases of necrotizing pneumonia and 2 pericarditis. One had a pyomyositis and another died of cardiac tamponade. One developed a chronic osteomyelitis and suffered from functional impairment.

## 4 Discussion

The clinical cases described in our study have in common a particularly severe presentation with a very high CRP and sometimes signs of septic shock with pneumonia or pericarditis. An extension of the infection or the formation of abscesses were also characteristic. Patient 4, who had a particularly severe plurifocal infection, has been transferred to a university reference hospital in Martinique, for orthopedic and cardiac care. Despite a favorable clinical course, the patient still has motor sequelae. Patient 1 died of tamponade during the pericardial puncture. These results are consistent with published studies.^[10–14]^ The MRI thus plays an important role in the diagnosis but especially in the preoperative assessment to estimate the extent of the infection. As stated in most studies, intravenous empiric antibiotics against SA and its toxins, given after a early and aggressive surgical management, seem to be a key point in the treatment.^[10–14]^ Indeed, drainage and washes repetition is often necessary to overcome the infection. But in our study, only one child underwent multiple surgeries. These SA-LPV infections can be life-threatening and are at high risk of complications and sequalae. The comparison with 4 studies,^[7,11–13]^ publishing clinical cases like ours, shows similarities, with the only difference that, unlike our study, there was no death. Even though French Guiana, which is a European region, is located in South America, our study reported a considerably lower proportion of MRSA. In the United States, the proportion of PVL-producing SA was consistently higher in infections caused by MRSA (74%–100%) than those caused by MSSA (9%–46%).^[15]^ The studies performed outside the United States, except in Greece, that included osteoarticular infections caused by both MSSA and MRSA, have reported a considerably lower proportion of MRSA.^[16]^ The high rate of MRSA is not linked to severity. Indeed, Gillet et al demonstrated that severity is linked with PVL secretion more than with resistance.^[7]^

Our results have some limitations: first, we know neither the number of PVL-positive cases that are treated in primary institutions nor the proportion of SA strains referred for PVL testing. We can thus expect that the number of BJI due to PVL-positive SA in French Guiana is currently probably underestimated. One might wonder if these infections would not be more common in tropical environments, where climatic factors might favor them. However, there was no information regarding the patients’ living conditions. Second, the rate of MRSA in the pediatric population seems very low in French Guiana. If the prevalence of PVL-producing strains depends on the prevalence of MRSA, this absence of MRSA in our study might play a role in the distribution of PVL in our region.

## 5 Conclusion

BJI due to SA-PLV seems to be more severe. In front of a severe acute osteomyelitis or arthritis, it is essential to look for the toxin, to perform MRI to estimate the extent of the infection. If not recognized, such infection can progress to septic shock, or even the death of the patient. Intravenous empiric antibiotics against SA and its toxins, given after an early and aggressive surgical management, seem to be a key point in the treatment. Given the high risk of complications, multidisciplinary management including aggressive surgical treatment is necessary.

## Author contributions

**Conceptualization:** Narcisse Elenga, Coralie Hardy, Lindsay Osei.

**Data curation:** Coralie Hardy, Lindsay Osei, Thierry Basset.

**Investigation:** Narcisse Elenga, Coralie Hardy.

**Methodology:** Narcisse Elenga, Thierry Basset.

**Supervision:** Narcisse Elenga.

**Validation:** Narcisse Elenga, Lindsay Osei.

**Visualization:** Thierry Basset.

**Writing – original draft:** Coralie Hardy.

**Writing – review and editing:** Narcisse Elenga, Lindsay Osei, Thierry Basset.

## References

[1] Iliadis AD Ramachandran M Paediatric bone and joint infection. EFORT Open Rev 2017;2:7–12. 28607765 10.1302/2058-5241.2.160027 PMC5444236

[2] Osei L El Houmami N Minodier P Paediatric bone and joint infections in French Guiana: a 6 year retrospective review. J Trop Pediatr 2017;63:380–8. 28204806 10.1093/tropej/fmw102

[3] Dohin B Gillet Y Kohler R Pediatric bone and joint infections caused by Panton-Valentine leukocidin-positive Staphylococcus aureus. Pediatr Infect Dis J 2007;26:1042–8. 17984813 10.1097/INF.0b013e318133a85e

[4] Bocchini CE Hulten KG Mason EO Jr Panton-Valentine leukocidin genes are associated with enhanced inflammatory response and local disease in acute hematogenous Staphylococcus aureus osteomyelitis in children. Pediatrics 2006;117:433–40. 16452363 10.1542/peds.2005-0566

[5] Otto M Staphylococcus aureus tsoxins. Curr Opin Microbiol 2013;17:32–7. 24581690 10.1016/j.mib.2013.11.004 PMC3942668

[6] Spaan AN van Strijp JAG Torres VJ Leukocidins: staphylococcal bi-component pore-forming toxins find their receptors. Nat Rev Microbiol 2017;15:435–47. 28420883 10.1038/nrmicro.2017.27 PMC5621924

[7] Gillet Y Dohin B Dumitrescu O Osteoarticular infections with staphylococcus aureus secreting Panton-Valentine Leucocidin. Arch Pediatr 2007;14 Suppl. 2:S102–7. 17956817 10.1016/s0929-693x(07)80043-1

[8] Labbé JL Peres O Leclair O Acute osteomyelitis in children: the pathogenesis revisited? Orthop Traumatol Surg Res 2010;96:268–75. 20488146 10.1016/j.otsr.2009.12.012

[9] Saavedra-Lozano J Falup-Pecurariu O Faust SN Bone and joint infections. Pediatr Infect Dis J 2017;36:788–99. 28708801 10.1097/INF.0000000000001635

[10] Mitchell PD Hunt DM Lyall H Panton-Valentine leukocidin-secreting Staphylococcus aureus causing severe musculoskeletal sepsis in children. A new threat. J Bone Joint Surg Br 2007;89:1239–42. 17905965 10.1302/0301-620X.89B9.19485

[11] Didisheim C Dubois-Ferrière V Dhouib A Severe osteoarticular infections with Staphylococcus aureus producer of Panton-Valentine Leukocidine in children. Rev Med Suisse 2014;10:355–9. 24624630

[12] Machuca MA González CI Sosa LM Methicillin-resistant Staphylococcus aureus causes both community-associated and health care-associated infections in children at the Hospital Universitario de Santander. Biomedica 2014;34 Suppl. 1:163–9. 24968048 10.1590/S0120-41572014000500019

[13] Albiński MK Lutz N Ceroni D Paediatric musculoskeletal infections with Panton-Valentine leucocidin. Swiss Med Wkly 2018;148:w14669. 30378636 10.4414/smw.2018.14669

[14] Gijón M Bellusci M Petraitiene B Factors associated with severity in invasive community-acquired Staphylococcus aureus infections in children: a prospective European multicentre study. Clin Microbiol Infect 2016;22: 643.e1-6. 10.1016/j.cmi.2016.04.004 27107685

[15] Ritz N Curtis N The role of Panton-Valentine leukocidin in Staphylococcus aureus musculoskeletal infections in children. Pediatr Infect Dis J 2012;31:514–8. 22327874 10.1097/INF.0b013e31824f18cb

[16] David MZ Daum RS Community-associated methicillin-resistant Staphylococcus aureus: epidemiology and clinical consequences of an emerging epidemic. Clin Microbiol Rev 2010;23:616–87. 20610826 10.1128/CMR.00081-09 PMC2901661

